# Development of tissue-engineered tracheal scaffold with refined mechanical properties and vascularisation for tracheal regeneration

**DOI:** 10.3389/fbioe.2023.1187500

**Published:** 2023-06-06

**Authors:** Tehreem Khalid, Luis Soriano, Mark Lemoine, Sally-Ann Cryan, Fergal J. O’Brien, Cian O’Leary

**Affiliations:** ^1^ School of Pharmacy and Biomolecular Sciences, RCSI University of Medicine and Health Sciences, Dublin, Ireland; ^2^ Tissue Engineering Research Group, RCSI University of Medicine and Health Sciences, Dublin, Ireland; ^3^ Advanced Materials and Bioengineering Research (AMBER) Centre, RCSI and Trinity College Dublin, Dublin, Ireland; ^4^ Centre for Research in Biomedical Devices (CÚRAM), NUI Galway, Galway, Ireland; ^5^ Trinity Centre for Biomedical Engineering, Trinity College Dublin, Dublin, Ireland

**Keywords:** trachea, tissue engineering, 3D printing, respiratory, vascularisation

## Abstract

**Introduction:** Attempted tracheal replacement efforts thus far have had very little success. Major limiting factors have been the inability to efficiently re-vascularise and mimic the mechanical properties of native tissue. The major objective of this study was to optimise a previously developed collagen-hyaluronic acid scaffold (CHyA-B), which has shown to facilitate the growth of respiratory cells in distinct regions, as a potential tracheal replacement device.

**Methods:** A biodegradable thermoplastic polymer was 3D-printed into different designs and underwent multi-modal mechanical assessment. The 3D-printed constructs were incorporated into the CHyA-B scaffolds and subjected to *in vitro* and *ex vivo* vascularisation.

**Results:** The polymeric backbone provided sufficient strength to the CHyA-B scaffold, with yield loads of 1.31–5.17 N/mm and flexural moduli of 0.13–0.26 MPa. Angiogenic growth factor release (VEGF and bFGF) and angiogenic gene upregulation (KDR, TEK-2 and ANG-1) was detected in composite scaffolds and remained sustainable up to 14 days. Confocal microscopy and histological sectioning confirmed the presence of infiltrating blood vessel throughout composite scaffolds both *in vitro* and *ex vivo*.

**Discussion:** By addressing both the mechanical and physiological requirements of tracheal scaffolds, this work has begun to pave the way for a new therapeutic option for large tracheal defects.

## 1 Introduction

Replacement of large tracheal defects is a complex issue, with little clinical success to date in spite of other advancements in modern medicine. Two key issues facing tracheal replacement are 1) the mechanical differences between the implant and surrounding native tissue and 2) of host acceptance of implants and integration into the host vascular network. Many promising studies have investigated novel approaches to replacing tracheal defects using additive manufacturing techniques (Les et al., 2019; Kang et al., 2019; Ricardo et al., 2019; Xia et al., 2019; Lee et al., 2020). The most notable were the Marlex™ mesh porous prosthesis, these showed promise in animal studies with 16 months of graft survival and patency (Beall et al., 1962). However, they did not perform well in humans due to stenosis and erosion of surrounding blood vessels (Maziak et al., 1996). Tracheal allografts have also been investigated since 1979 (Rose et al., 1979). Recent developments by Delaere et al. (2012), Delaere et al. (2014), and Delaere et al. (2019) saw the development of a two-step technique in which the donor trachea is revascularised in the patient’s forearm before implantation. A cadaveric trachea was decellularised, implanted in the forearm of the recipient and seeded with buccal mucosa to enhance vascularisation prior to implantation (Delaere et al., 2010). This approach was further explored with four additional patients, but tracheal necrosis and poor vascularisation hampered the outcome of this clinical trial (Omori et al., 2008). 3D-printing has also been utilised to treat tracheal defects in paediatric patients as 3D printed airway splints have been granted FDA approval (Pacetti, 2020). First reported in 2013 to treat a severe case of tracheobronchomalacia (TBM) (Zopf et al., 2013), the splints have been successfully implanted in a wider set of patients with low mortalities (Les et al., 2019; Morrison1 et al., 2017; Huang et al., 2016; Morrison et al., 2017). However, their use has been restricted to paediatric patients. Only one attempt has been reported in an adult, with maintained airway patency 3-month post implantation. Overall, there is currently no established gold standard for tracheal regeneration that fully addresses all major concerns.

The mechanical discrepancy between native tracheal tissue and that of the replacement graft is often one of the major causes for implant failure *in vivo* and within clinical efforts (Grimmer et al., 2004; Azorin et al., 2006; Luo et al., 2013; Wood et al., 2014; Elliott et al., 2017). The trachea is composed of distinct structural regions that each fulfill a distinct role in tracheal stability, as well as respond differently to mechanical stimuli, which collectively gives the trachea its unique anisotropic mechanical properties (Teng et al., 2012). The trachea’s horse-shoe shaped hyaline cartilage rings, smooth muscle and annular ligament, collectively produce an anisotropic tissue that allows for longitudinal extensibility and lateral rigidity (Teng et al., 2008; Teng et al., 2012; Boazak and Auguste, 2018). Therefore, any tracheal substitute must be mechanically robust in order to withstand intra-thoracic pressure changes that occur during respiration (Rose et al., 1979; Maziak et al., 1996). Conversely, they must also be able to deform radially to allow for changes in the cross-sectional area during coughing and swallowing (Boazak and Auguste, 2018). These complicated native tissue characteristics, coupled with a lack of standardised protocols to accurately quantify tracheal biomechanics as guidance for implant design, constitute a significant hurdle for tracheal biomaterial scaffold fabrication.

Heterogenous methods to characterise native tracheal mechanics include different specimen types, shapes, testing methods, and parameters suffer from inconsistency [reviewed in Soriano et al. (2021)]. This lack of consistency is likely due to the geometry of the trachea, together with its unique structural regions, which make it difficult to define the most appropriate mechanical tests to perform to best capture the correct biomechanics (Lambert et al., 1991; Rains et al., 1992; Roberts et al., 1997; Hoffman et al., 2016; Siddiqi, 2017). The inability to collectively define the mechanical properties of the trachea has resulted in studies simply using uniaxial compression testing alone to characterise their scaffolds, which upon *in vivo* implantation, have subsequently failed due to the mismatch in mechanical properties provoking graft collapse or dislodgement (Grimmer et al., 2004; Wood et al., 2014). To this end, we propose to establish a benchmark of different mechanical test types that are able to evaluate the bulk radial compressive properties of our scaffold but also their flexibility and resistance to applied static load over time, ultimately to recapitulate the mechanical environment of the trachea.

3D-printing (3DP) provides the capacity to generate complex and custom designs that enable the fabrication of patient-specific constructs. Furthermore, due to its reproducibility and the ability to alter mechanical properties of printed constructs through the fine-tuning of complex design features, 3DP was selected in this study to fabricate a polymeric tubular framework for a composite tracheal scaffold. To generate a structure that can produce a tracheal scaffold with mechanical support similar to native cartilage rings, polycaprolactone (PCL) was selected as the printing material due its strong mechanical properties, non-cytotoxic degradation products and long-term stability (Sun et al., 2006; Woodruff and Hutmacher, 2010) These properties have meant that the use of 3DP PCL in tracheal replacement efforts has been widespread, with studies fabricating tracheal scaffolds that display excellent resistance to compressive stresses and facilitate cartilage tissue formation (Gao et al., 2017; Best et al., 2018; Les et al., 2019; Xia et al., 2019; She et al., 2021). However, 3DP PCL constructs *in vivo* have also suffered from poor results due to inflammatory responses that cause granulation tissue formation and stenosis (Lin et al., 2009; Gao et al., 2017; Rehmani et al., 2017; Kaye et al., 2019; Xia et al., 2019). Notably, in a recent study of a 3D-printed scaffold coated in a chondrocyte suspension and implanted within a rabbit model, all animals had died by 10 weeks, with 75% of deaths occurring linked to excess granulation tissue. Furthermore, no re-epithelisation was found to have occurred (Kaye et al., 2019). Thus, while PCL holds great potential as a base material for mechanically robust tracheal scaffolds, additional components are needed to augment the construct’s integration to its surrounding mucosal and submucosal tissue.

The other key issue facing tracheal replacement is that of vascularisation of the implant. This is a critical consideration to avoid tissue necrosis in the implant site and to support new cartilage and epithelium formation (Walles et al., 2004; Li et al., 2019). Tracheal revascularisation—or, more precisely, the lack thereof—was deemed to be a key factor in a high prolife case of scientific misconduct in this field, which involved the implantation of solid polymer tubes seeded with stem cells as tracheal replacement (Delaere and Van Raemdonck, 2016; Delaere et al., 2019). These studies highlighted the importance of the trachea’s blood supply, and the need for any substitute graft to effectively support revascularisation and ensure clinical success. Pre-vascularisation prior to implantation has shown higher survival rate of animals, lower rates of stenosis and increased re-epithelisation, suggesting the presence of an established vascular network enhances graft survivability (Luo et al., 2013; Maughan et al., 2017; Wu et al., 2017; Bae et al., 2018). Indeed, our in-house approach to engineer a prevascularised within collagen scaffolds with co-culture of human umbilical vein endothelial cells (HUVECs) and human mesenchymal stem cells (hMSCs) provided *in vitro* and *in vivo* stable vessel formation (Hoffman et al., 2016).

Therefore, we propose that the integration of a 3DP PCL framework within a pre-vascularised collagen matrix can enhance implant integration into surrounding tracheal tissue, as a biocompatible substrate for successful cellular attachment and growth. In this study, building on our bilayered collagen-hyaluronic acid (CHyA-B) matrix (O’Leary et al., 2016), our objective was to incorporate a 3DP PCL framework to produce a reinforced composite CHyA-B scaffold, and demonstrate successful pre-vascularisation as a proof-of-concept prior to future preclinical animal studies.

## 2 Materials and methods

### 2.1 Design and manufacture of 3D-printed PCL framework

**FIGURE 1:**
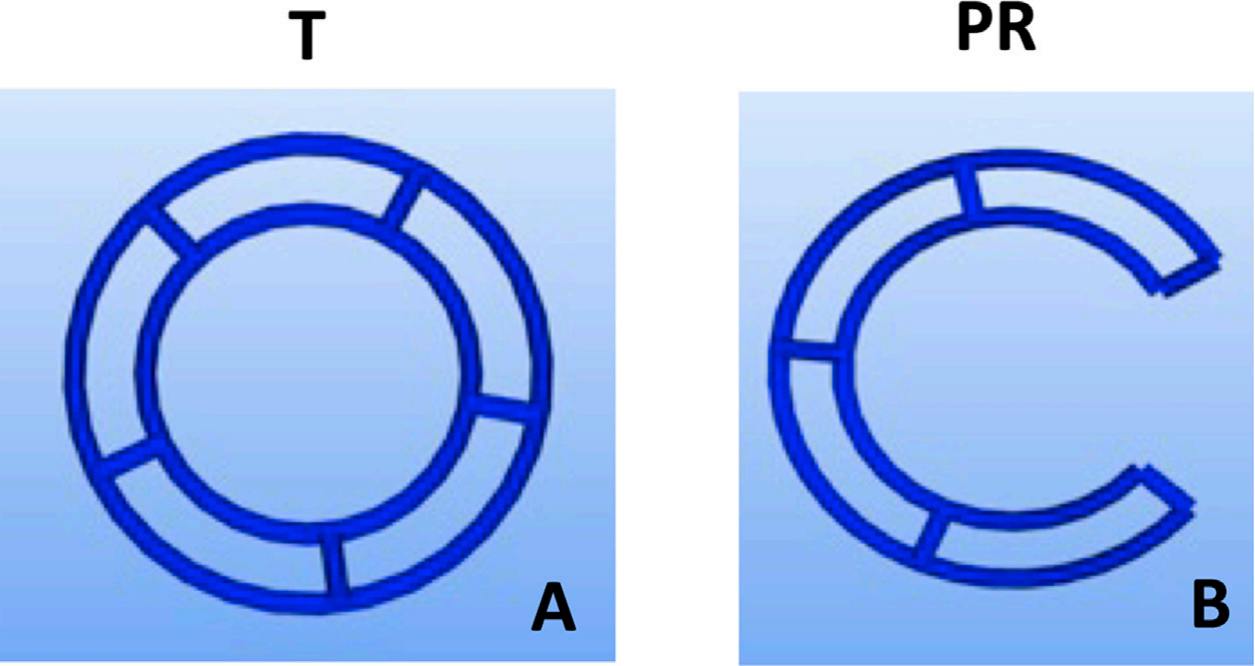
Design iterations of 3DP tracheal scaffolds. Two main design features were fabricated: Tubular T; **(A)** and Partial Ring PR; **(B)**.

Fused deposition modelling 3D-printing was utilised to fabricate novel tubular frameworks with the aim to optimise printing parameters and to mechanically characterise scaffolds for tracheal replacement. The frameworks were designed using custom G-code developed from Python programs, which contain toolpaths for the print head, to generate a design with either a complete 360° tubular ring (T) or 288° partial ring (PR). Both designs featured two rings, 2 mm apart, connected by five struts (Figure 1; Table 1). The designs were 3D printed using an Allevi II Bioprinter (Allevi, United States) with 25 kDa PCL (Polysciences, Germany). PCL was heated to 90°C and extruded at a pressure of 60 psi at a printing speed of 260 mm/min. All designs were printed using a 25G metal needle (Micron-S, Fisnar, ECT Adhesives, Ireland).

**TABLE 1:** Dimensions and structural differences of tracheal scaffold designs.

Scaffold Design	Dimensions (Inner Ring Radius, mm)	Dimensions (Outer Ring Radius, mm)	Opening (degrees)	Number of Struts
Tubular (T)	4.8	6.8	—	5
Partial Ring (PR)	4.8	6.8	72	5

### 2.2 Mechanical testing

#### 2.2.1 Radial compression

To determine the mechanical strength of the different designs and the influence of molecular weights of PCL, radial compression was performed using a universal mechanical testing machine Z005 (Zwick/Roell, German, A702416) with a 50 N load cell. The 3DP frameworks were compressed radially at an applied strain of 50% at 0.6 mm/min. The yield point was calculated from the load-deformation curves using the 0.2% offset method. The partial ring designs (PR1-3) were tested in two different testing positions: lateral (L) and anterior-posterior (A-P) to assess the influence of the opening gap on mechanical strength.

#### 2.2.2 Cyclical testing

To assess the durability of the different designs, the 3DP frameworks were subjected to cyclical testing. With the universal mechanical testing machine and 50 N load cell, the strain rate was adjusted to mimic a fast-breathing rate of adult humans at 20 breaths per minute for 250 cycles. The designs were placed vertically onto the machine and fine grit sandpaper was placed between the framework and the testing platens to prevent slippage. The peak load at cycles 1 and 250 was measured and the percentage cyclical strain recovery was calculated to elucidate the design’s ability to recovery to its original shape. The percentage of strain recovery after 250 cycles was determined using Eq. 1, where *ε_m_* is the applied strain and *ε_p_(N)* is the strain after N cycles (Lendlein et al., 2001).

$$R_{r}=\frac{\left(\varepsilon_{m}-\varepsilon_{p}\left(N\right)\right)}{\varepsilon_{m}}$$ (1)

#### 2.2.3 Three-point bend testing

To examine the flexibility of the designs, a 3-point bending rig was used to assess the bending stiffness of the frameworks. In order to provide comparable data, flexural moduli of the scaffolds were assessed as seen in literature for tracheal scaffolds (Park et al., 2018). Frameworks were compressed at 50% strain until failure with the universal mechanical testing machine and 50 N load cell. The partial ring designs were again assessed in two different orientations, firstly with the opening facing down towards the lower plate and secondly with the opening facing out sideways, to assess the influence of the gap on the flexibility of the design.

### 2.3 Manufacture and characterisation of reinforced composite CHyA-B scaffolds

#### 2.3.1 Collagen-hyaluronate slurry and film preparation

Collagen-hyaluronic acid (CHyA) slurry and films were prepared using a previously described method (O’Leary et al., 2016). In short, 0.5% w/v collagen (type 1 collagen, bovine tendon) (Collagen Solution, United Kingdom) and 0.044% hyaluronic acid (Hyaluronic acid sodium salt from *Streptococcus* equi; Sigma-Aldrich, Arklow, Ireland) in 0.5 M acetic acid were homogenously mixed using an Ultra Turrax T18 Overhead Blender (IKA Works Inc., United States) at 15,000 rpm at 4°C for 3.5 h, and subsequently degassed to 4,000 mTorr under a vacuum to produce a CHyA slurry, which was stored at 4°C. To manufacture CHyA films, 50 mL of the CHyA slurry was cast into 12.5 cm^2^ × 12.5 cm^2^ stainless steel moulds clamped to a polytetrafluoroethylene (PTFE) plate and dehydrated overnight under a constant air flow to produce a thin and transparent CHyA film.

#### 2.3.2 Reinforced composite CHyA-B scaffold manufacture

**FIGURE 2:**
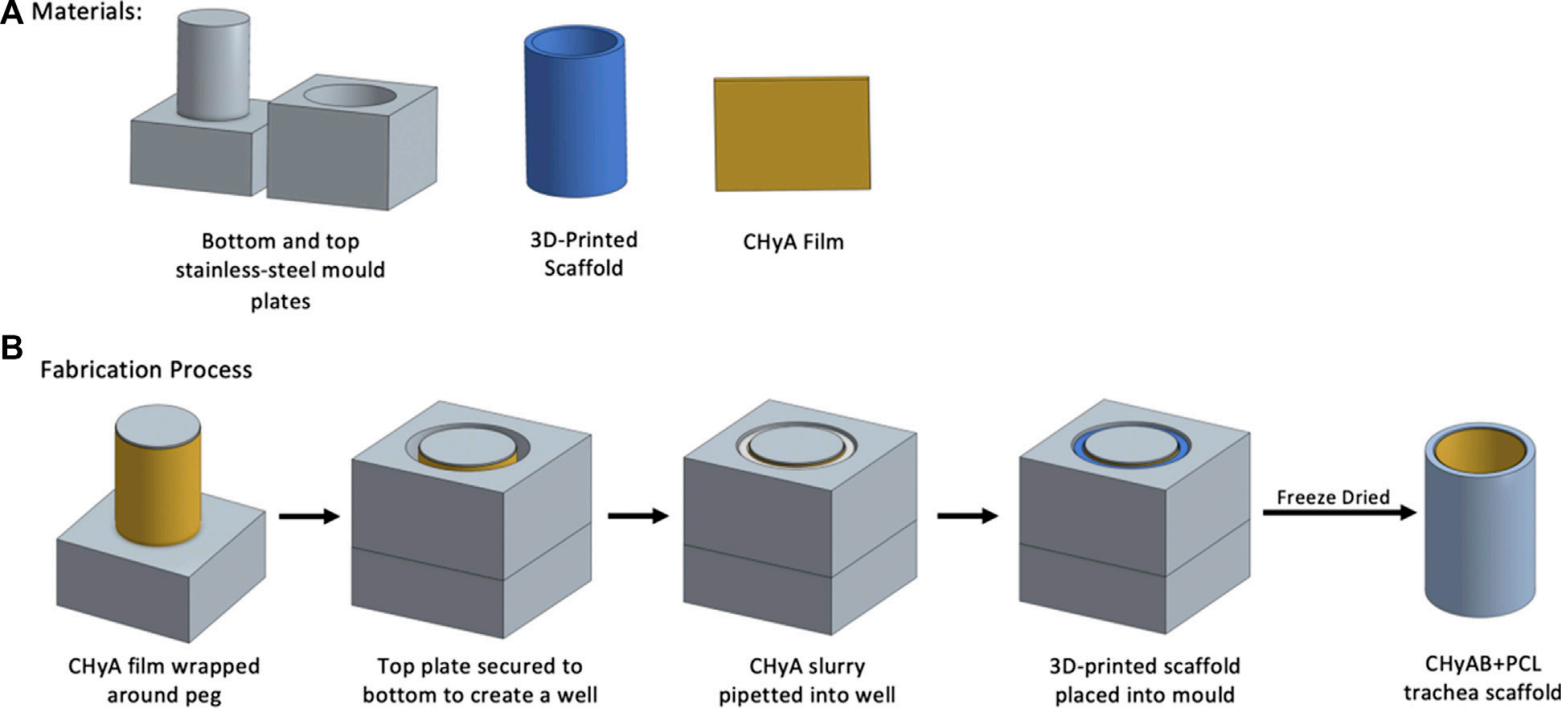
Schematic overview of fabrication of reinforced composite CHyA-B tracheal scaffolds. **(A)** Materials required for scaffold fabrication. **(B)** Fabrication process of reinforced composite CHyA-B tracheal scaffolds. In brief, CHyA films are wrapped around the pegs of the bottom mould plates and top plates is secured on forming a well in which CHyA slurry is pipetted into. Following this, the 3D-printed frameworks are placed into the mould and freeze dried to form a reinforced composite CHyA-B tubular scaffold.

Prior to incorporating the 3D-printed frameworks to CHyA slurry for freeze-drying, the constructs were surface treated with 3 M sodium hydroxide (NaOH) for 48 h at room temperature, and then rinsed three times with diH20 to remove any residual NaOH. Following the surface treatment, a custom stainless-steel mould was used to fabricate the reinforced composite CHyA-B scaffolds (Figure 2). The mould consists of a bottom plate with 16 pegs, 26 mm in height and 7.8 mm in diameter and a top plate with 14 mm wide circular holes. First, CHyA films were cut into strips of 26 mm × 30 mm and rehydrated in 0.5 M acetic acid for 1 h at room temperature. Upon rehydration, the films were wrapped around the pegs of the bottom plate and left overnight under a constant air flow to dehydrate the films, enabling them to firmly attach to the metal pegs. The following day, the top plate of the mould was then secured on, creating a well in which 1,750 μL of CHyA slurry was pipetted into and then 3D-printed frameworks were then gently placed into the slurry. Subsequently, the films were allowed to rehydrate in the collagen slurry within the mould for 2 h and then freeze-dried using a customised anneal freeze drying cycle. The scaffolds were subjected to an initial freezing step to −20°C for 1 h in which ice crystals formed within the collagen slurry. Following this, the temperature was raised to −10°C, an annealing step, which merged the ice crystals resulting in larger pores. Finally, to allow for sublimation and drying of the scaffolds, the pressure was decreased to 200 mTorr.

### 2.4 Scaffold ultrastructure

Reinforced composite CHyA-B scaffolds were examined using scanning electron microscopy (SEM) in order to evaluate the collagen integration and the ultrastructure between the PCL fibres. Samples were mounted to an aluminium stub and sputter-coated with gold/palladium (Cressington 108auto, Cressington Scientific Instruments, United Kingdom) at a current of 40 mA for 80 s. Imaging of the scaffolds was performed using a Zeiss Ultra Plus scanning electron microscope (Zeiss, Germany). Images were captured at 5 kV using the secondary electron mode. Three scaffolds from two batches of reinforced CHyA-B scaffolds were used for imaging.

### 2.5 Pore size analysis

Having established the 3DP design and mechanical properties of the scaffolds, the scaffold’s ability to support cell infiltration and growth was assessed by measuring the mean pore size of the collagen sub-layer of composite scaffolds. Pore size of the reinforced composite and non-reinforced CHyA-B matrix were analysed to assess the effect of the incorporating tubular 3D-printed framework on the mean pore size of CHyA-B matrices. Samples of reinforced CHyA-B scaffolds were cut into 1 cm × 1 cm sections and analysed using a technique optimised for pore size analysis of collagen scaffolds (O’Brien et al., 2005). In short, the scaffolds were dehydrated using a series of increasing concentrations of ethanol and then embedded in JB-4^®^ glycolmethacrylate (Polysciences Europe, Eppelheim, Germany). Sections of 10 μm thickness of embedded samples were sectioned using a carbon steel blade (C35, PFM, Laboratory Instruments and Supplies, Ireland) on a microtome (Leica RM 2255, Leica, Germany). Sections were stained using 0.5% toluidine blue for 4 min and imaged at ×10 magnification using a microscope (Eclipse 90i, Nikon, Japan), with attached camera (DS Ri1, Nikon, Japan). Captured images were analysed using a custom-made MATLAB (MathWorks Inc., MA, United States) script developed by our group in conjunction with the Sigmedia Research Group in the Electrical Engineering Department at Trinity College Dublin, Ireland. Images were converted to binary form (black and white) and using a best fit of elliptical lengths the average pore diameter was measured. For each sample, sections were taken every 100 μm spanning the entire length of the scaffold and three scaffolds of each group from two batches of freeze-drying were analysed.

### 2.6 HUVEC and hMSC co-culture on reinforced composite CHyA-B scaffolds

#### 2.6.1 Scaffold fabrication for cell culture

**FIGURE 3:**
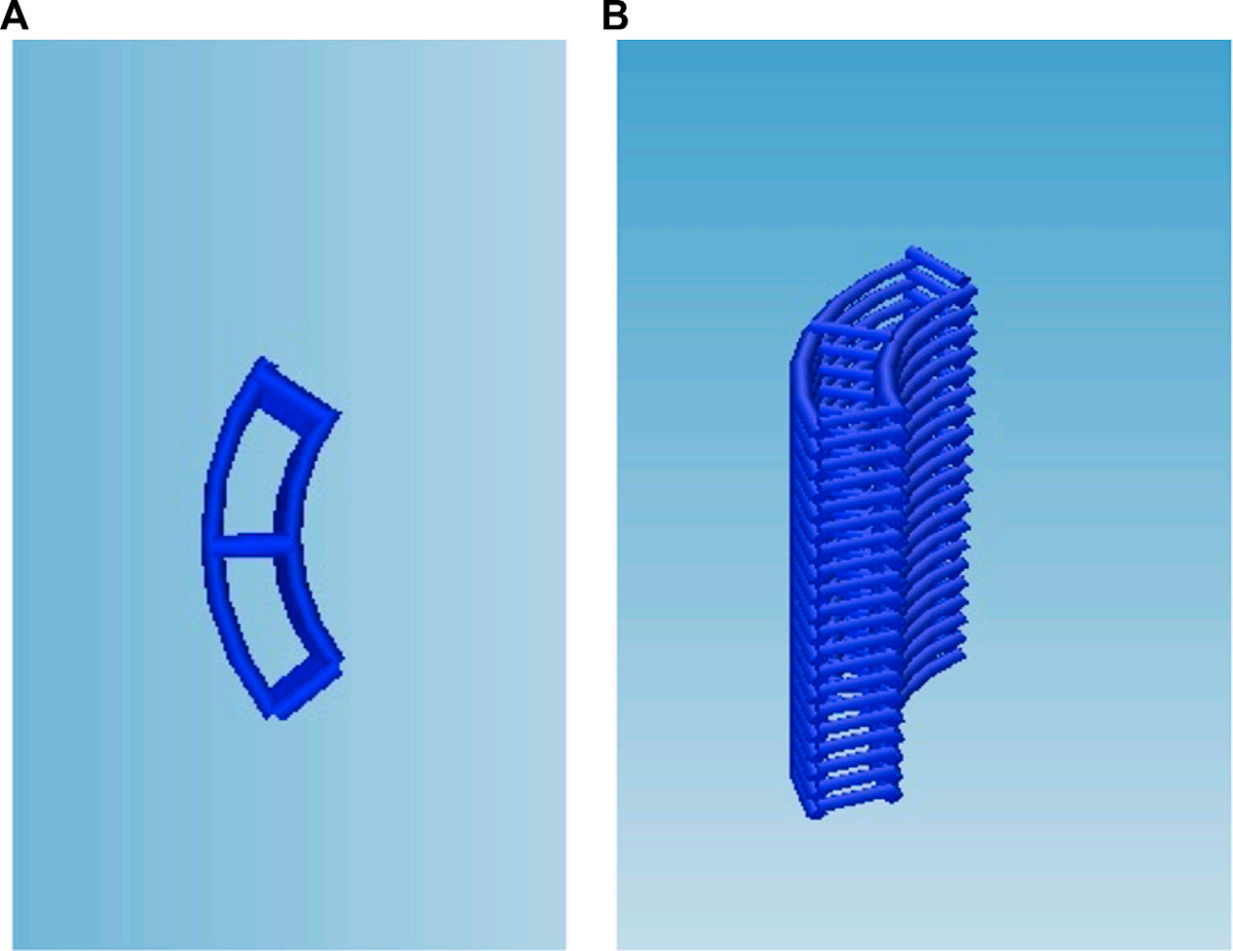
Scaffold design for cell culture study, the g-code script was altered to reduce the circumference and height of the scaffold to retain the **(A)** strut as **(B)** a stacked 6 × 6 mm scaffold.

In order to biologically characterise the reinforced composite CHyA-B scaffolds, 6 mm × 6 mm sections were 3D-printed as previously described (Section 2.3) and incorporated and freeze-dried with CHyA slurry and film (Section 2.3.2; Figure 3). Non-reinforced CHyA-B matrices were used as control and were sectioned from a tubular non-reinforced CHyA-B matrices.

#### 2.6.2 Sterilisation and crosslinking

All scaffolds for cell study were placed under 254 nm UV light for 30 min on each side for sterilisation at a distance of 10 cm before being chemically cross-linked using 1-ethyl-3-(3-dimethylaminopropyl) carbodiimide hydrochloride (EDAC) (Haugh et al., 2011; Lemoine, 2020). Scaffolds were firstly pre-hydrated in Dulbecco’s Phosphate Saline (DPBS) before being submerged into a solution of 6 mM EDAC per gram of collagen was mixed with N-hydroxysuccinimide (NHS) at a molar ratio of 2.5 M EDAC: 1 M NHS in 70% ethanol. The scaffolds were then washed three times with sterile DPBS to remove residual cytotoxic product. Scaffolds were stored at 4°C for up to a week after crosslinking or used immediately for *in vitro* experiments. All steps were performed under sterile conditions.

#### 2.6.3 Cell sources and media

All cells present in this study were obtained from Lonza. hMSCs were originally isolated from bone marrow aspirates obtained from the iliac crest of healthy human donors by the commercial supplier with ethical approval (Lonza, Switzerland). HUVECs were commercially acquired (pooled, CC-2719; Lonza, Switzerland). Cells were cultured at 37°C and 5% CO2 under humidified atmosphere. hMSC growth medium corresponded to low glucose (1 g/L) Dulbecco’s modified Eagle’s medium supplemented (LG-DMEM, Sigma-Aldrich, Ireland) with 10% foetal bovine serum (FBS; Biosera, Ringmer, United Kingdom) and 1% penicillin/streptomycin. HUVEC growth medium corresponded to endothelial growth medium-2 (EGM-2, Lonza, Switzerland). Medium was replaced every 3 days, and upon reaching 80%–90% confluence, cells were passaged using trypsin-EDTA solution. For all experiments described below, the highest passage number used for both cell types were passage 5.

#### 2.6.4 Scaffold seeding

To evaluate the influence of 3DP PCL framework on *in vitro* vascularisation of reinforced composite CHyA-B scaffolds, a previously optimised vascularisation cell culture model was utilised, compromising of a co-culture of HUVECs and hMSCs at a ratio of 4:1, respectively (McFadden et al., 2013). All chemically crosslinked and sterilised scaffolds were preconditioned overnight in EGM-2 prior to seeding. Scaffolds were then seeded in a delayed stepwise manner on their porous collagen sub-layer, using 25 μL of cell suspension containing 2 × 105 HUVECs in a dropwise manner. The samples were subsequently incubated for 1 h to allow for cell attachment, followed by the addition of EGM-2. Three days post-HUVEC seeding, scaffolds were seeded with 5 × 104 hMSCs in 25 μL, using the same approach.

### 2.7 Cell viability

To assess and compare cellular viability of the co-culture cells on scaffolds, a battery of tests was performed over a 14-day period using assays. An alamarBlue™ metabolic assay (Invitrogen, BioSciences, Ireland) and PicoGreen dsDNA assay (Invitrogen) were performed at days 6, 10 and 14 of culture, in addition to fluorescent imagining of live and dead cells at day 14 using LIVE/DEAD^®^ Viability/Cytotoxicity Kit (Thermofisher, Ireland) all according to the manufacture’s protocol.

### 2.8 Vessel formation in reinforced composite CHyA-B scaffolds

#### 2.8.1 Immunofluorescence

To image the formation of vessel-like structures within scaffolds, scaffolds were harvested at days 6, 10 and 14, for fluorescence labelling (do Amaral et al., 2019a). The scaffolds were washed with PBS and fixed in 10% neutral buffered formalin solution (Sigma-Aldrich, Ireland) for 1 h at room temperature. Following fixation, scaffolds were washed three times with PBS and cut in half transversely to expose the cross-section for imaging. 0.3% Triton X-100 in PBS followed by 3% bovine serum albumin (BSA; Sigma-Aldrich, Ireland) in PBS were applied for 20 min each to permeabilise cells and block unspecific antigen binding. Monoclonal mouse anti-human CD31 antibody (Dako, M0823) diluted to 1:50 in 1% BSA in PBS was used for HUVEC identification and a rabbit polyclonal antibody for α-smooth muscle actin (α-SMA; Abcam, ab5694) diluted at 1:100 in 1% BSA in PBS was applied to detect hMSCs. Primary antibodies were incubated overnight at 4°C. Rat anti-mouse IgH (H + L) secondary antibody, Fluorescein isothiocyanate (FITC; Biosciences, Ireland, 11401185), diluted at 1:100 in 1% BSA in PBS and goat anti-rabbit IgG (H + L) cross-adsorbed secondary antibody, Alexa Flour 633 (Invitrogen, A-21070), diluted at 1:400 in 1% BSA in PBS was applied for 1 h at room temperature. Thereafter, 1:600 diluted Phalloidin–Tetramethylrhodamine B isothiocyanate (TRITC; Sigma-Aldrich, Ireland) in 1% BSA in PBS was applied for 20 min to stain the cell cytoskeleton followed by 4′, 6-Diamidino-2-phenlindole dihydrochloride (DAPI; Sigma-Aldrich, Ireland) at 1:1,000 dilution in PBS for 20 min for nuclei staining. Samples were stored in PBS at 4°C until imaging with a Carl Zeiss LSM 710 confocal microscope with a W N-Achroplan 10x (numerical aperture 0.3) lens. Z-stack images were taken at 30 μm depths below the surface to yield a total 150 μm total depth.

#### 2.8.2 Chick chorioallantoic membrane (CAM) model

To assess the influence of the 3DP PCL framework within the CHyA-B matrix on vessel infiltration *ex vivo*, scaffolds were incubated within a chick chorioallantoic membrane (CAM) model as previously described (do Amaral et al., 2019a; Ryan et al., 2019; do Amaral et al., 2019b). Fertilised chicken eggs (Ovagen Group Ltd., Co. Mayo, Ireland) were purchased at day 0 of development and incubated at 37°C for 3 days. Following incubation, the eggs were cracked into 100 mm diameter petri dishes (Corning Inc., New York, United States), which in turn were then placed within larger 150 mm diameter petri dishes containing sterile PBS, to form a humidified chamber. The chicks were incubated for a further 4 days, completing 7 days of total development, viability of embryos was firstly ensured and checked at every day from this point forward; when cross-linked and sterilised CHyA-B matrices and reinforced composite CHyA-B scaffolds were placed within the chick membrane and incubated for a further 5 days, allowing for vessel ingrowth. At day 12 the samples were harvested and imaged. The vascularisation around the scaffolds was quantified using ImageJ software after treatment with the “Mexican Hat Filter” to outline the blood vessels, and then converted to 8-bit and the blood vessel area was measured.

#### 2.8.3 Histology

Harvested scaffolds from CAM were stained with haematoxylin and eosin (H&E) to observe vessel infiltration throughout the scaffold. Scaffolds were fixed overnight in 10% neutral buffered formalin solution at 4°C. Following fixation, scaffolds were dehydrated in increasing concentrations of ethanol until 100% ethanol was reached. Scaffolds were then placed within xylene solution under agitation, to dissolve out PCL filaments, after which they were processed using an automated tissue processor (ASP300, Leica, Germany) for paraffin infiltration. The samples were then embedded into paraffin blocks and were sectioned as described in Section 2.5 to obtain sections with a thickness of 7 μm in their cross-section to observe vessels infiltration. Sections were rehydrated in decreasing concentrations of ethanol, and then incubated with Harris haematoxylin (Sigma-Aldrich, Ireland) for 5 min before being washed in tap water for 5 min. Differentiation of the samples was then performed with acidified 70% ethanol, before being stained with 0.1% eosin Y (Sigma-Aldrich, Ireland) in 95% ethanol. Finally, slides were dehydrated and mounted with DPX. Images were captured as described in Section 2.5.

#### 2.8.4 Pro-angiogenic protein expression

The expression of pro-angiogenic markers vascular endothelial growth factor (VEGF) and basic fibroblast growth factor (b-FGF) were quantified using ELISA kits (R&D, Biotechne, Ireland). Supernatants on days 6, 10 and 14 were collected and analysed following the manufacturer’s protocol.

#### 2.8.5 Pro-angiogenic gene expression

To assess gene expression within scaffolds seeded with the co-culture model, scaffolds were harvested and stored at −80°C in 1 mL of Qiazol lysis reagent (Qiagen, Crawley, United Kingdom). Total RNA was isolated using a RNeasy Mini Kit according to manufactures protocol (Qiagen). RNA quality and quantity was determined using Nanodrop 2000 Spectrophotometer (Thermofisher, Ireland). Reverse transcription of RNA lysates was performed on 400 ng of total RNA using the QuantiTect Reverse Transcription kit (Qiagen) according to manufacturer’s protocol on a MiniAmp Thermal Cycler (A37834, Thermofisher). Quantitative real time polymerase chain reactions were performed in duplicate on a 7500 Real-Time PCR system (Applied Biosystems) using QuantiTect SYBR Green PCR kit (Qiagen). The mRNA relative expression was calculated by 2^−ΔΔCT^ method, in which CHyA-B matrices were used as control relative to each respective timepoint. Target mRNAs analysed were KDR (QT00069818, Qiagen), ANG1 (QT00046865, Qiagen), ANG2 (QT00100947, Qiagen), TEK2 (QT01666322, Qiagen), FLT1 (QT00270823, Qiagen) and 18S (QT00199367, Qiagen) was used as a housekeeping gene.

### 2.9 Data analysis

Quantitative data were analysed using GraphPad Prism 8.0 software. Two-tailed student t-tests were used for statistical analysis between two groups, and 1-way or 2-way ANOVA was used for statistical analysis between multiple groups. Sidak’s multiple comparison *post hoc* analysis was performed in all ANOVA assessment.

## 3 Results

### 3.1 Scaffold characterisation

#### 3.1.1 Mechanical testing

**FIGURE 4:**
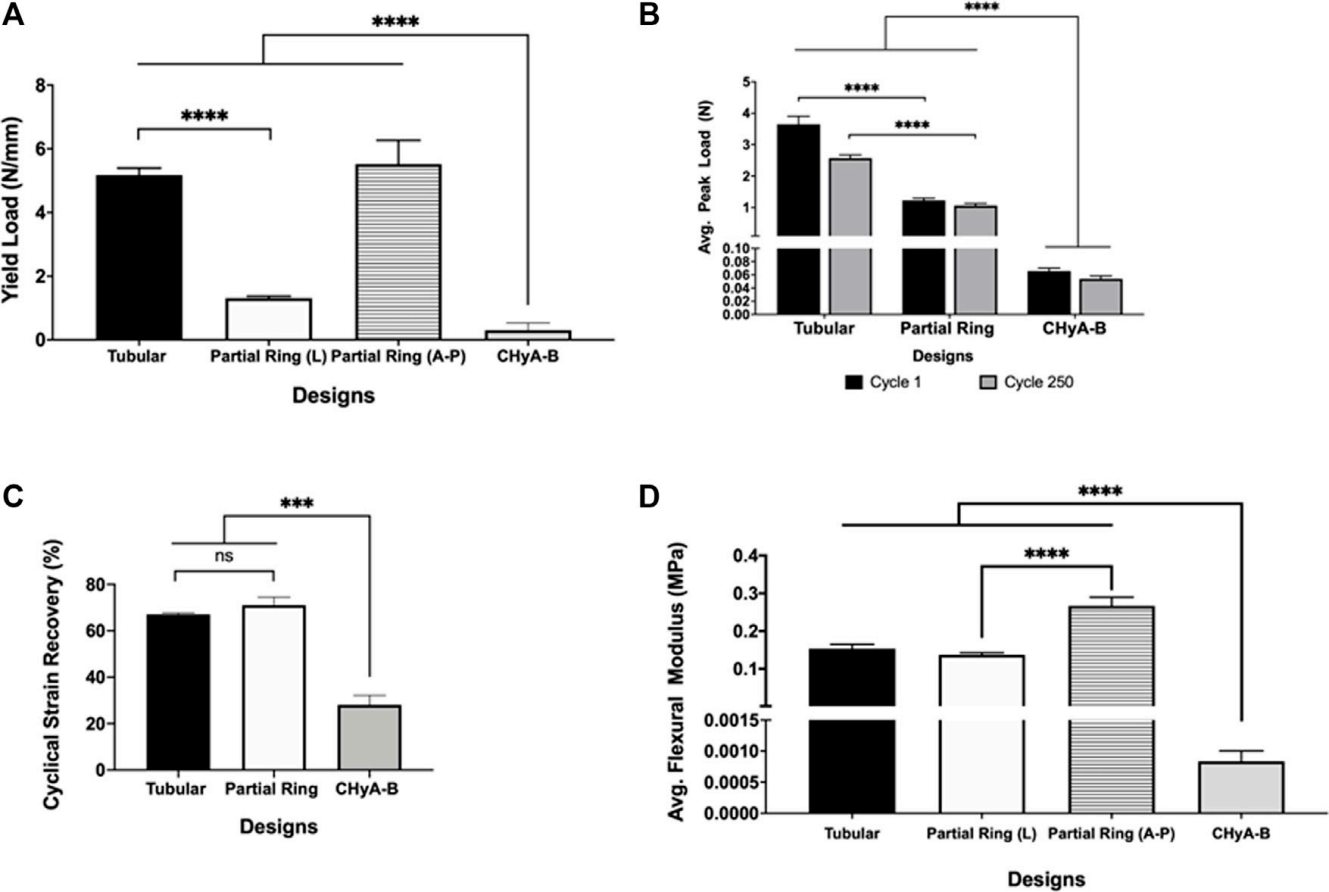
Mechanical properties of 3DP tracheal framework designs. Scaffolds were subjected to multimodal mechanical assessment to compare the influence of different scaffold designs. **(A)** Compression testing, **(B, C)** cyclical testing, **(D)** Three-point bending. Results displayed as mean ± SEM, *n* = 4. ns = *p* > 0.05, ***p < 0.001, ****p < 0.0001.

To determine the mechanical strength of the two designs, uni-axial compressive testing was carried out (Figure 4). A significant increase in the yield load was calculated in the tubular design, 5.177 ± 0.219 N/mm, and the A-P and L partial-ring designs, 5.523 ± 0.744 N/mm and 1.313 ± 0.061 N/mm respectively, compared to CHyA-B matrices, 0.307 ± 0.453 N/mm. Notably, a difference in yield load was observed between the two orientations of the PR design, suggesting this design better recapitulated the anisotropic mechanical properties found in native tracheal tissue.

Moreover, to assess the durability of the different designs, and whether the 3D printed scaffolds could withstand the constant mechanical loading and unloading that the trachea experiences during respiration, all scaffold designs underwent cyclical testing. Irrespective of design feature, all scaffold designs withstood cyclical loading over 250 cycles and remained patent, with no visible cracks or failure sites observed when inspected visually. However, a significant 2N difference in average peak load was observed between tubular and partial ring design at cycle 1, which reduced to a 1N difference at cycle 250, highlighting the influence of the partial ring design when under radial mechanical stresses (Figure 4B). Nonetheless, the difference in structural design did not influence the percentage cyclical strain recovery (Figure 4C). Scaffolds recovered an average of 67% of the applied strain following 250 cycles to 15% applied strain in the tubular design and 71% in the partial ring design, significantly greater than the non-reinforced CHyA-B matrix.

To determine the flexibility of the scaffolds, both designs underwent three-point bend testing. The partial ring design was tested under two different orientations in relation to the position of the opening gap (Figure 4D). The tubular design exhibited a higher flexural modulus of 0.153 ± 0.010 MPa. Additionally, the partial ring design also exhibited a similar high flexural modulus in the lateral position (0.131 ± 0.005 MPa) but was significantly more flexible in the second testing position A-P (0.266 ± 0.022 MPa).

#### 3.1.2 Scaffold ultrastructure

**FIGURE 5:**
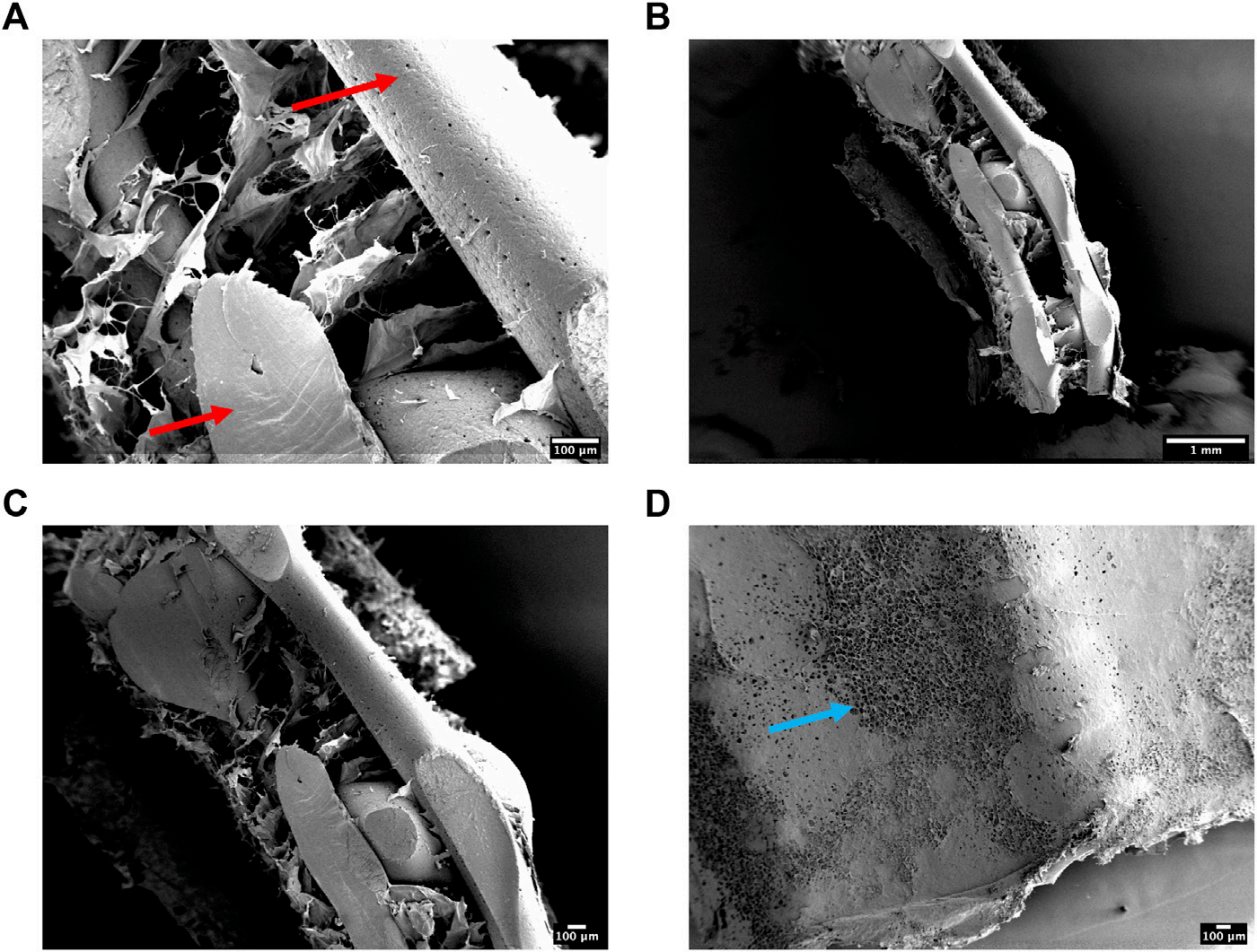
**(A–C)** Scanning electron micrographs showing the ultrastructure of cross-sectioned reinforced composite CHyA-B scaffolds. PCL indicated with red arrow. **(D)** A representative surface image of the non-film outer collagen surface layer showing the porosity (blue arrow) of the collagen sub-layer. *n* = 2, in triplicate.

Having successfully mechanically characterised the 3DP designs, we next sought to evaluate the ultrastructure of the reinforced composite CHyA-B scaffolds after incorporating the 3DP frameworks into CHyA-B matrices using SEM. Scaffolds were imaged using SEM to assess the influence of the PCL fibres and scaffold design on collagen matrix infiltration. Subsequent SEM assessment captured successful integration of collagen within the PCL fibres (Figure 5). Therefore, a favourable structure was achieved through a reproducible process achieving successful integration of a porous collagen network around a 3D-printed backbone structure.

#### 3.1.3 Pore size analysis

**FIGURE 6:**
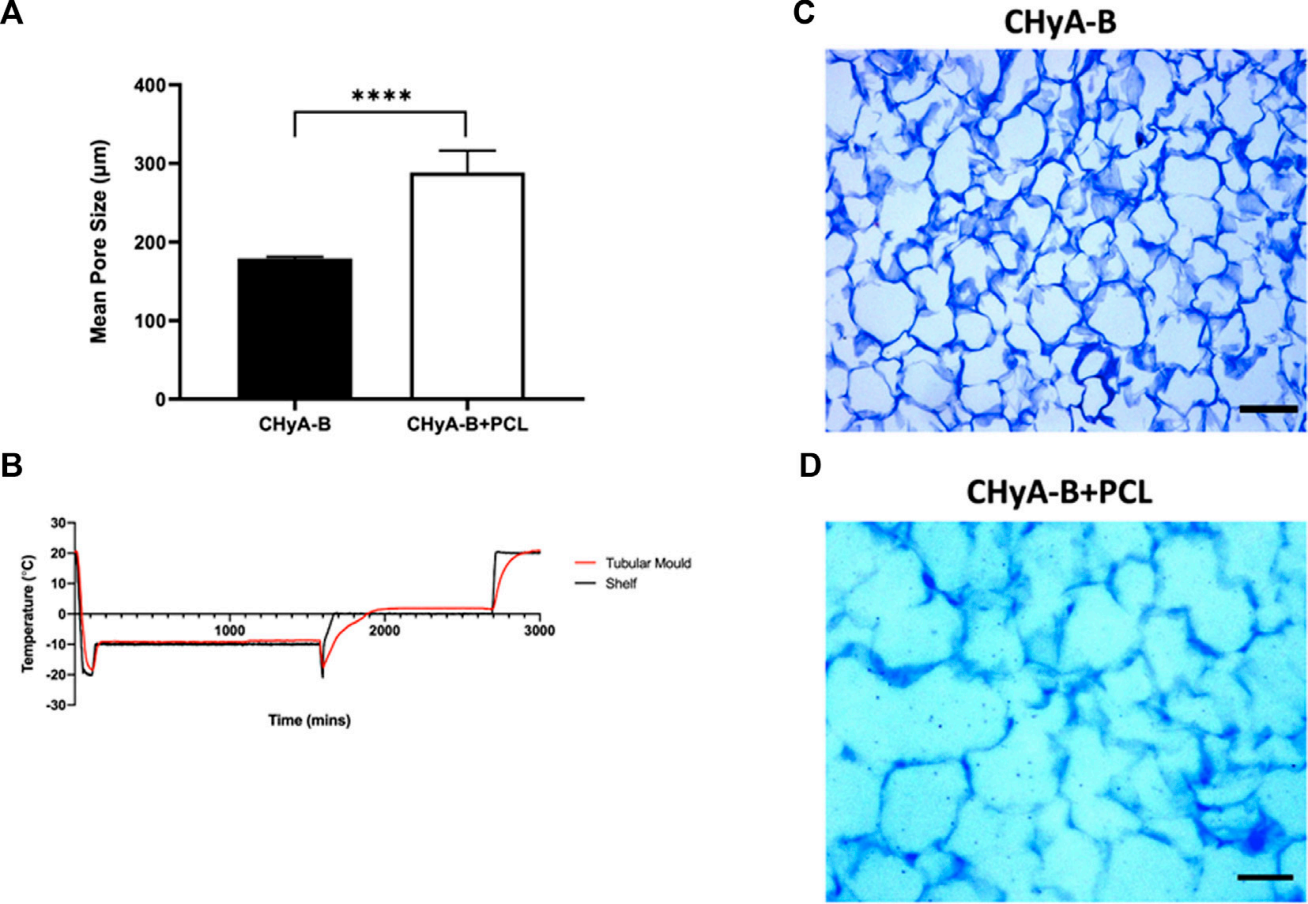
Pore size analysis of bilayered composite scaffold. **(A)** Mean pore diameter of non-reinforced (CHyA-B) matrices and reinforced composite (CHyA-B+PCL) scaffold. **(B)** Temperature profile of tubular moulds during −20 anneal cycle in comparison to shelf temperature, data captured by Dr. Derek Whelan, RCSI (*n* = 1). Toluidine blue sections of CHyA-B **(C)** and CHyA-B+PCL **(D)** scaffolds used for software analysis Scale bar = 100 μm. Results displayed as mean ± SEM, *n* = 3, in quadruple. ****p < 0.0001.

To assess the scaffold’s ability to support cellular infiltration and growth, the mean pore size of the collagen matrix in the sub-layer of reinforced composite CHyA-B scaffolds was analysed. The inclusion of the 3D-printed PCL framework within CHyA-B tubular matrix resulted in an average pore size of 290 ± 27.66 µm compared to an average pore size of 180 ± 1.962 µm in non-reinforced tubular CHyA-B matrices (Figure 6A). Temperature probes placed within the tubular moulds and freeze-dryer shelf recorded differences in freeze drying profiles (Figure 6B), with the temperature within the tubular moulds requiring a longer time to be brought up back to temperature during the cycle, possibly influencing the pore size of the resultant scaffolds (Figures 6C, D).

### 3.2 Cell viability

**FIGURE 7:**
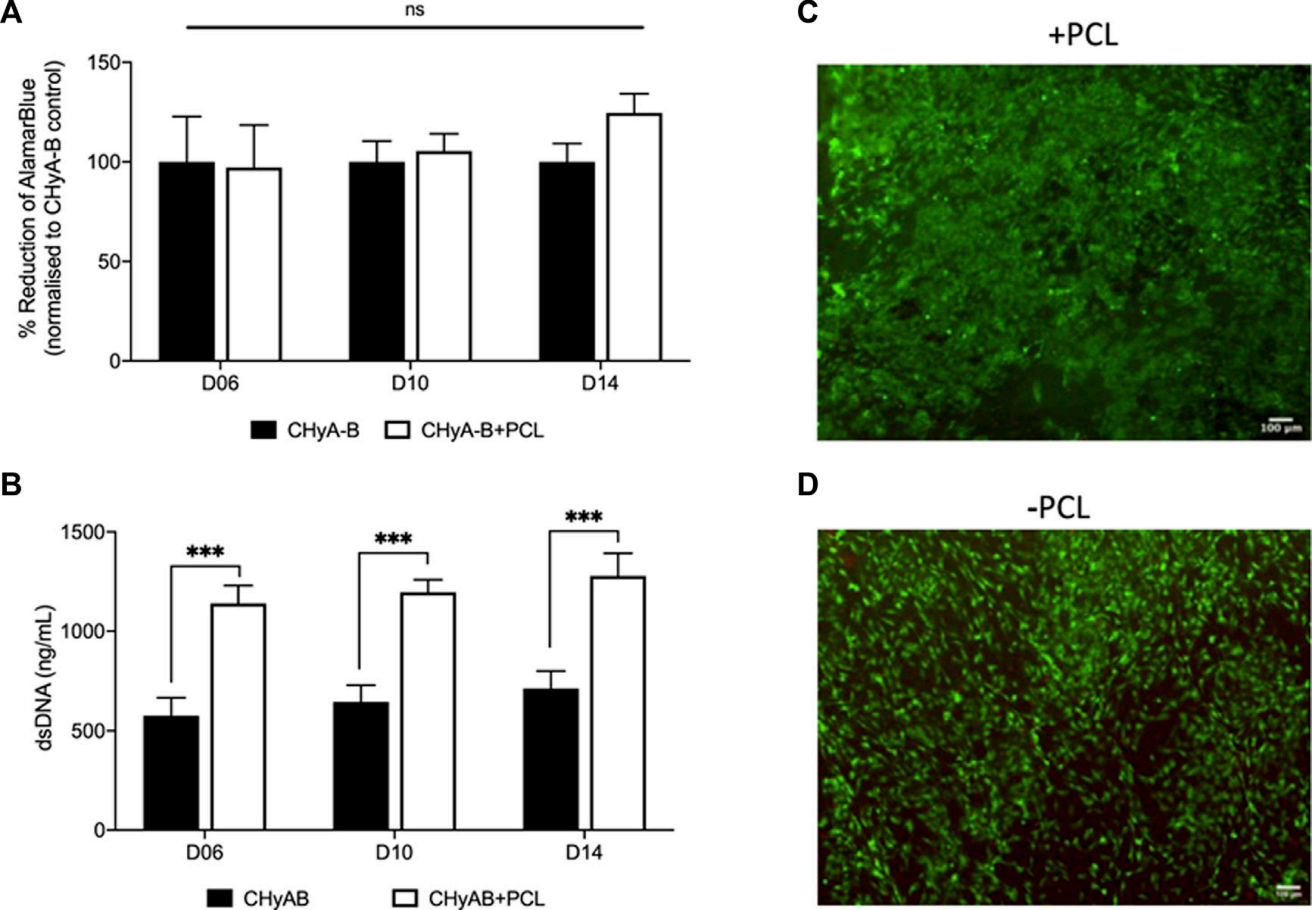
Assessment of cell viability of reinforced composite CHyA-B scaffolds. CHyA-B matrices and reinforced composite CHyA-B scaffolds were subjected to cell viability tests to assess their biocompatibility with HUVECs and hMSCs. **(A, B)** Cellular metabolic activity and double stranded DNA content measured in reinforced composite scaffolds (CHyA-B+PCL) and non-reinforced matrix (CHyA-B). Representative LIVE/DEAD^®^ images taken of the surface of CHyA-B **(C)** and CHyA-B+PCL **(D)** scaffolds at day 14 at ×4 magnification. Scale bar = 100 μm. Results displayed as mean ± SEM, *n* = 3, in triplicate. ns = *p* > 0.05, ***p ≤ 0.001.

In an aim to assess and compare cell viability of the vascularisation co-culture system on reinforced CHyA-B scaffold to that of CHyA-B matrix, scaffolds were subjected to multiple viability assays (Figure 7). Cell metabolic activity measured in reinforced composite CHyA-B scaffolds was comparable to measurements in control CHyA-B matrices over 14 days of culture period (Figure 7A). Notably, double stranded DNA (dsDNA) content was two-fold greater in reinforced composite CHyA-B scaffolds compared to CHyA-B matrices over 14 days of cell culture (Figure 7B). LIVE/DEAD^®^ imaging also confirmed the presence of a high population of green viable cells on both scaffold types (Figure 7C). Overall, the mix of quantitative and qualitative data indicate that the inclusion of 3DP PCL framework within CHyA-B matrices sustains vascular cell viability, and potentially facilitates increased proliferation.

### 3.3 Vessel formation in reinforced composite CHyA-B scaffolds

#### 3.3.1 Immunofluorescence

**FIGURE 8:**
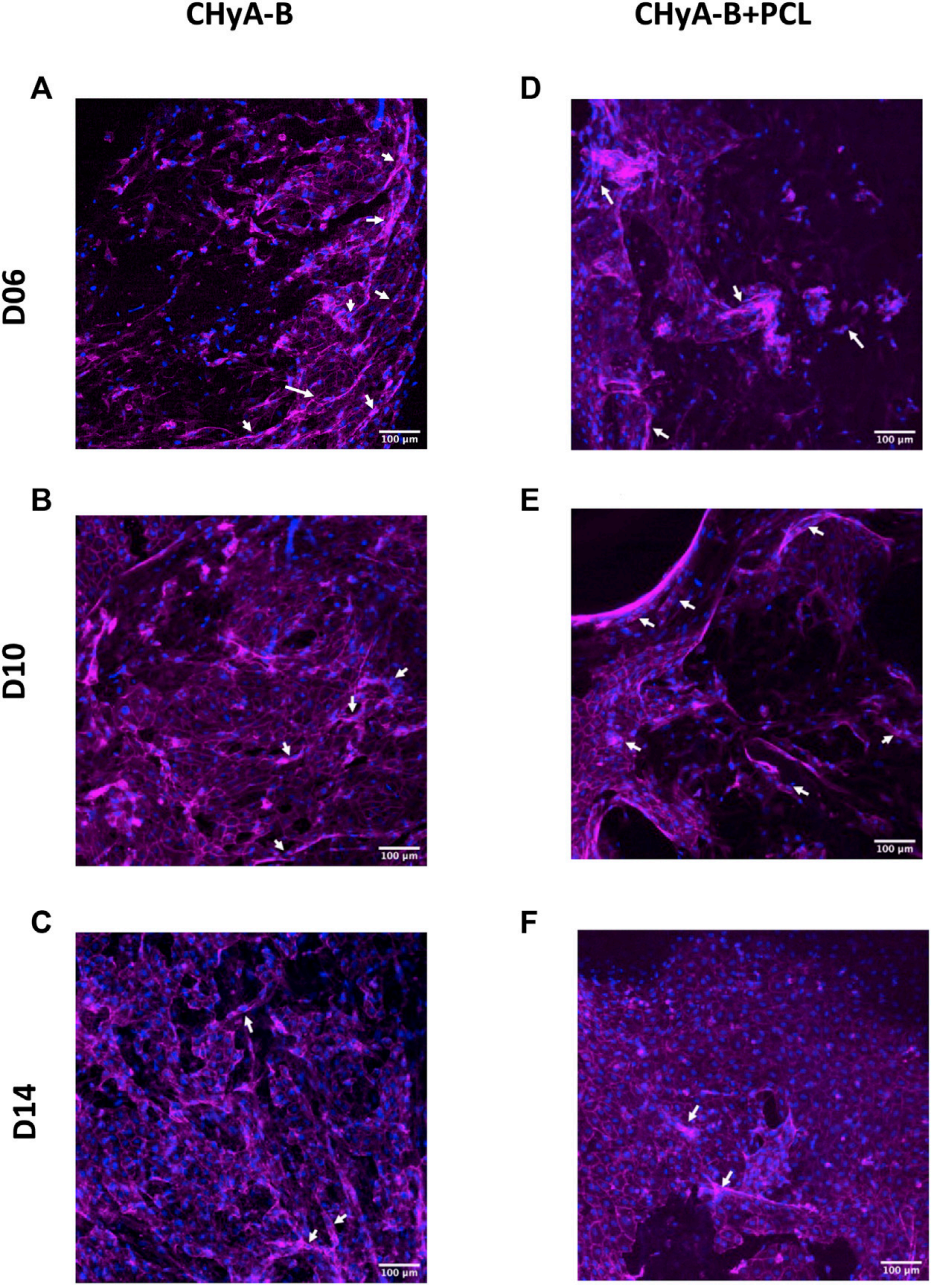
Actin cytoskeleton and nuclei staining on reinforced composite CHyA-B scaffolds and CHyA-B matrices *in vitro* vascularisation. HUVECs were cultured with hMSCs on either **(A–C)** non-reinforced matrices (CHyA-B) or **(D–E)** reinforced composite scaffolds (CHyA-B+PCL) scaffolds for 14 days. Maximum intensity projections reconstructed from z-stack images display cells arranged in tubule-like structures (white arrows). Magenta = f-actin, blue = nuclei. *n* = 3, in triplicate. Scale bar = 100 μm.

**FIGURE 9:**
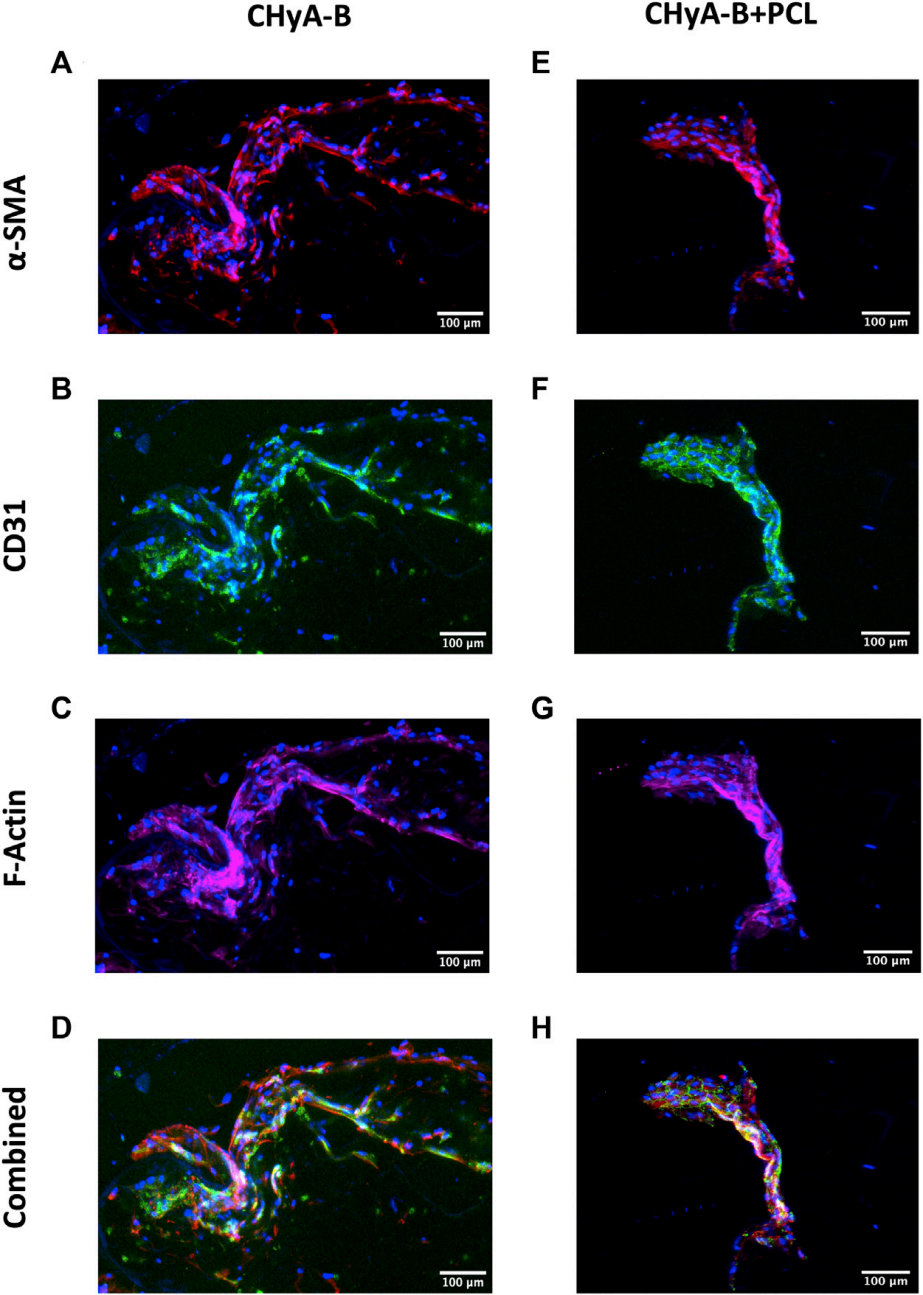
CD31 and α-SMA expression by HUVECs and hMSCs in co-culture on reinforced composite CHyA-B scaffolds and CHyA-B matrices at day 10 on *in vitro* culture. HUVECs were cultured with hMSCs on either **(A–D)** non-reinforced matrices (CHyA-B) or **(E–H)** reinforced composite scaffolds (CHyA-B+PCL). Maximum intensity projections reconstructed from z-stacks display tubule-like structures lined with HUVECs CD31 stained in green; **(B, F)** and hMSCs in proximity α-SMA stained in red; **(C, G)**. *n* = 3, in triplicate. Scale bar = 100 μm. Nuclei = blue, f-actin = magenta, α-SMA = red, CD31 = green.

To image the formation of vessel-like structures within scaffolds, cells were fluorescently labelled and then imaged using confocal microscopy. The presence of vessel-like structures was visible in reinforced composite CHyA-B scaffolds and CHyA-B matrices across 14 days of culture (Figure 8; white arrows), with fewer vessel-like structures on the post-peak timepoint of day 14. Furthermore, fluorescence labelling of endothelial cell makers CD31 and pericyte cell marker α-SMA on day 10 of cultured samples (Figure 9) confirmed the presence of both cell types around vessel-like tubules. Thus, we confirmed that the incorporation of 3DP PCL framework within CHyA-B matrix did not impede of vessel formation *in vitro*.

#### 3.3.2 Pro-angiogenic protein expression

**FIGURE 10:**
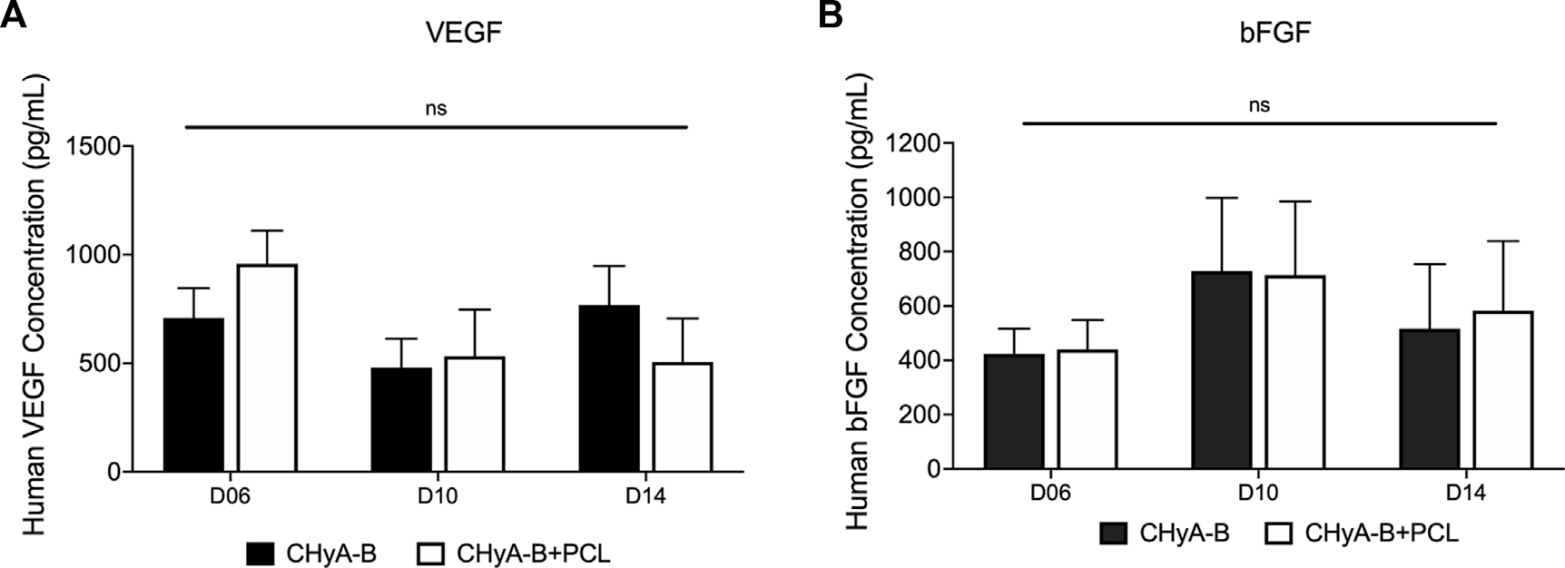
Expression of pro-angiogenic proteins **(A)** VEGF and **(B)** b-FGF were measured over 14-day culture period on scaffolds in co-culture of HUVECs and hMSCs on reinforced composite scaffolds (CHyA-B+PCL) and non-reinforced CHyA-B matrices. Results displayed as mean ± SEM. *n* = 3, in triplicate. ns = *p* > 0.05.

The expression of pro-angiogenic markers VEGF and b-FGF was quantified using ELISA kits on days 6, 10 and 14 of *in vitro* culture. Although no significant difference was observed between reinforced composite CHyA-B scaffolds and CHyA-B matrices; over the 14 days of culture period, comparable levels of VEGF and b-FGF was measured (Figure 10).

#### 3.3.3 Pro-angiogenic gene expression

**FIGURE 11:**
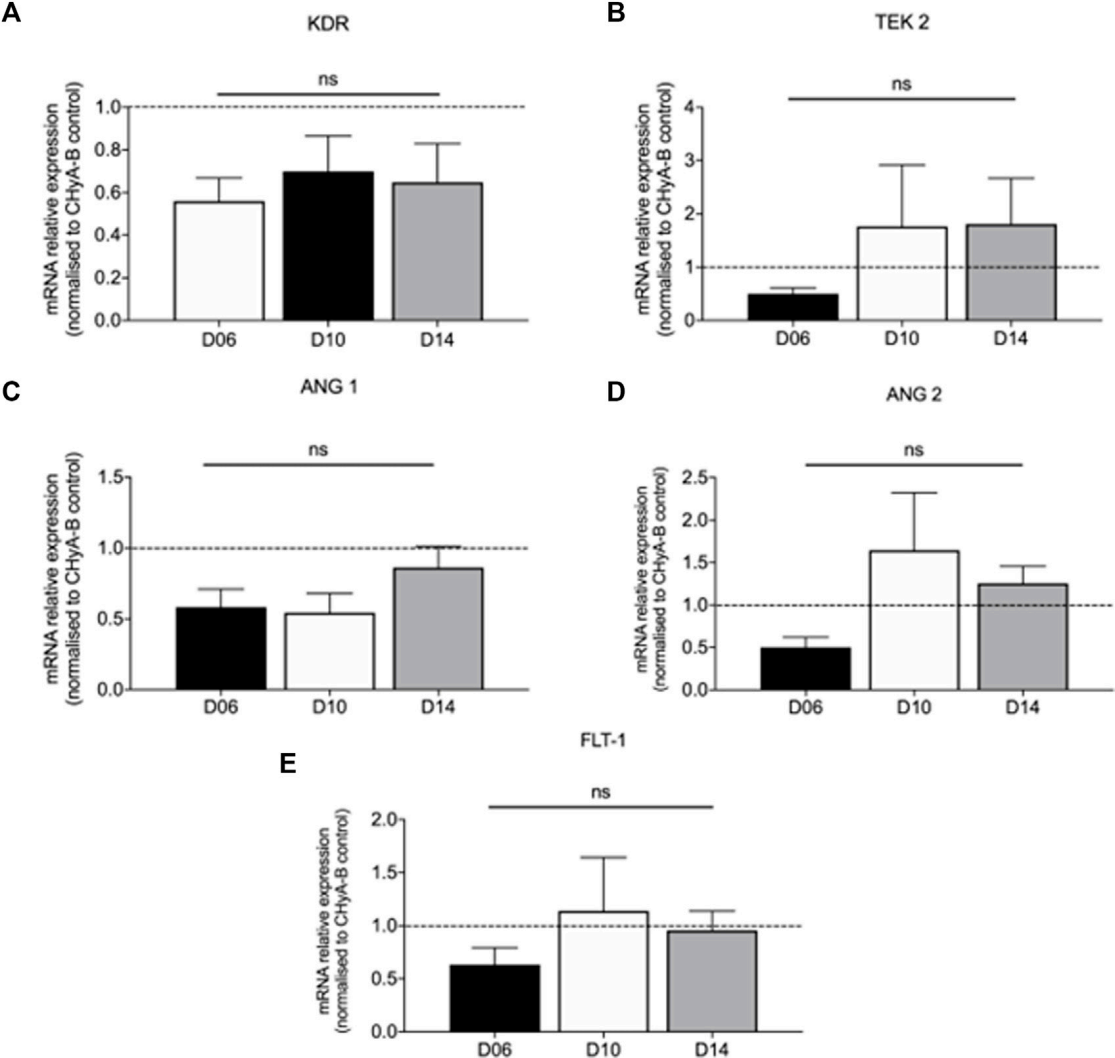
Expression of pro-angiogenic genes expressed in co-culture of HUVECs and hMSCs on reinforced composite CHyA-B+PCL scaffolds over 14-day culture period. Quantification of mRNA expression of **(A)** KDR expression **(B)** TEK 2 expression **(C)** ANG 1 expression **(D)** ANG 2 and FLT-1 expression **(E)** displayed as mean ± SEM with expression relative to mRNA expression in CHyA-B matrices at each relative time point. *n* = 3; in triplicate. ns = *p* > 0.05.

The influence of reinforced composite CHyA-B scaffolds on HUVEC and hMSC co-culture gene expression of KDR, TEK 2, ANG 1, ANG 2, and FLT 1 was analysed to assess the effect of the 3DP PCL framework on angiogenic gene expression. The inclusion of the 3DP PCL framework was found to not significantly alter the expression of angiogenic genes (Figure 11), with gene expression sustained over 14 days of *in vitro* culture. Taken together with the expression of pro-angiogenic proteins (Figure 10), this work demonstrates the mechanically-reinforced tracheal scaffold’s ability to sustain angiogenic marker expression *in vitro*.

#### 3.3.4 Chick chorioallantoic membrane (CAM) model

**FIGURE 12:**
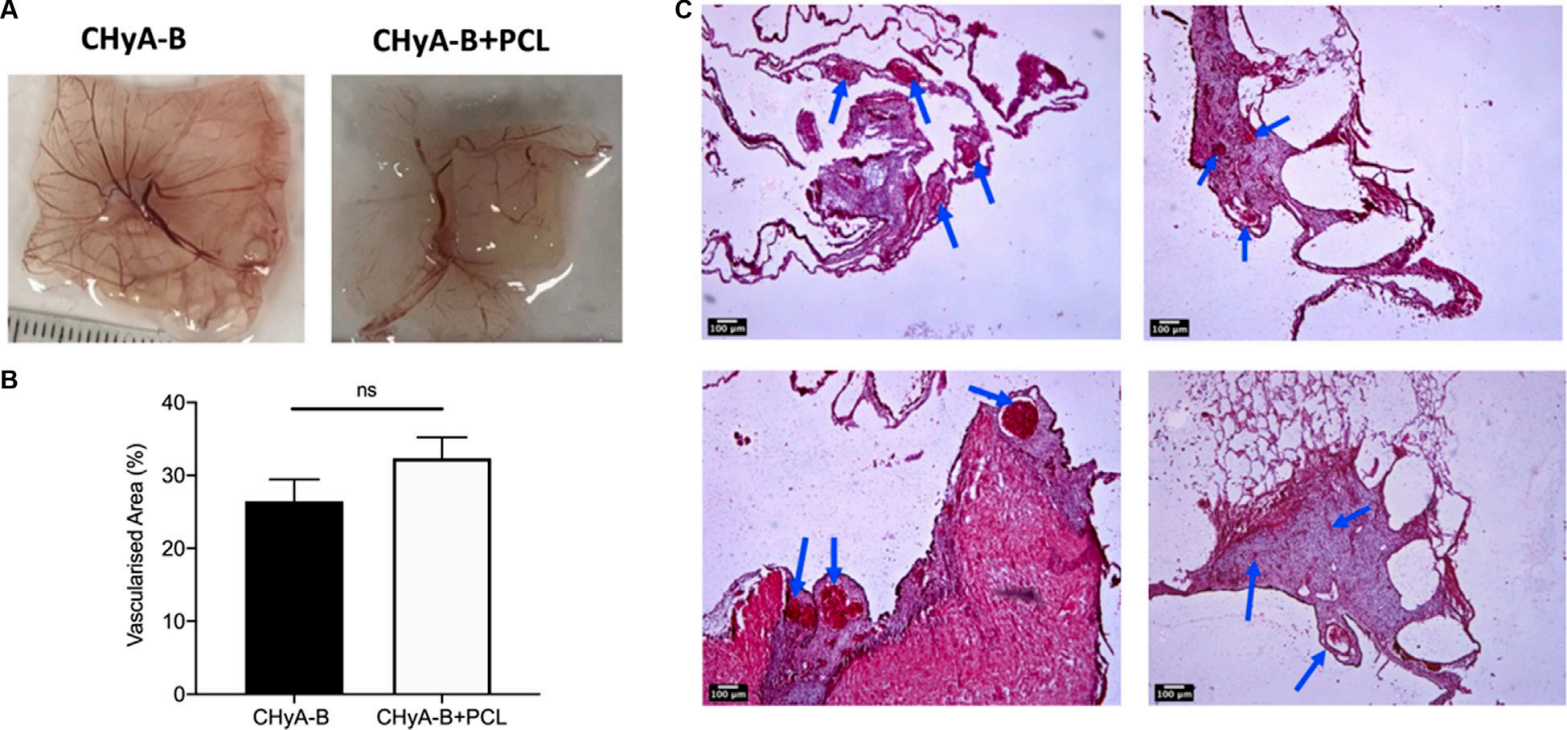
Reinforced composite CHyA-B scaffolds were vascularised *ex vivo* in a chick chorioallantoic membrane (CAM) model. **(A)** Macro-images taken of harvested scaffolds after incubation within CAM. **(B)** Image analysis of macro-images of harvested scaffolds calculated greater vascularised area within reinforced composite scaffolds (CHyA-B+PCL) than the non-reinforced CHyA-B matrices. **(C–F)** H&E stained sections of CHyA-B matrices **(C, D)** and reinforced composite CHyA-B+PCL scaffolds **(E, F)** imaged at ×4 magnification. Staining confirmed the presence of blood vessels infiltrating throughout the scaffolds as highlighted by blue arrows. Results displayed as mean ± SEM, *n* = 5. ns = *p* > 0.05. Scale bar = 100 μm.

To assess the influence of the 3DP PCL framework on vessel infiltration *ex vivo*, scaffolds were analysed using a CAM model, in which they were placed within fertilised chick eggs and incubated to allow for living blood vessels to infiltrate. Image analysis of macro-images of harvested samples (Figure 12A) calculated an increase in vascularised area in the reinforced composite CHyA-B scaffolds over its non-reinforced counterpart, although not statistically significant (Figure 12B). Furthermore, sectioned samples of reinforced composite scaffolds also confirmed the presence of vessels penetrating the scaffolds confirming that the incorporation of PCL fibres did not disrupt vessel infiltration (Figures 12C–F). These are important findings as we have shown the successful fabrication of a novel mechanically reinforced tubular scaffold for tracheal tissue regeneration, with the ability to successfully vascularise *in vitro* and *in vivo*, which would enhance graft survivability post implantation.

## 4 Discussion

In this study, we sought to fabricate a composite biomaterial-based scaffold for the regeneration of damaged tissue in large tracheal defects. The major objective of the study was to investigate the potential of fabricating reinforced CHyA-B scaffolds tubular constructs with similar mechanical properties to native tracheal tissue by incorporating a 3D-printed PCL framework within the porous collagen sub-layer. In particular, we assessed the feasibility of 3D-printing tubular constructs, evaluated the mechanical properties of different design, and validated their *in vitro* biocompatibility and then the ability to successfully vascularise. Multi-modal mechanical assessment led to the mechanical characterisation of the 3DP framework to achieve two lead designs (tubular and partial ring) with mechanical properties comparable to native tracheal tissue. Furthermore, the incorporation of the 3DP framework and tubular form-factor generated a scaffold with greater mean pore diameter, ideal for cellular infiltration. Biological characterisation of the reinforced composite CHyA-B scaffolds demonstrated their ability to support cellular growth. Additionally, vascularisation within the PCL-reinforced collagen matrix was maintained with no adverse outcome; vessel-like tubules were observed repeatability in scaffolds using a suite of methods. The expression of pro-angiogenic markers was also investigated, to examine the effect of 3DP PCL on protein and gene expression, with no significant difference observed to the standard vascularised scaffold controls. Finally, in the first step to *in vivo* validation of the scaffolds, the CAM model was utilised and confirmed sustained levels of vascularisation within the reinforced composite scaffolds compared to CHyA-B matrices. Collectively, the data presented within this chapter confirmed that the incorporation of 3DP PCL for mechanically reinforcing CHyA-B matrices maintained successful vascularisation within the collagen matrix and represented a step towards a functional, multi-cellular, and viable construct for tracheal regeneration. Taken together, the results demonstrate that a composite CHyA-B scaffold can be developed to recapitulate the mechanical properties of native tracheal tissue with no negative effects on cell proliferation and vascularisation.

Tracheal scaffold designs were conceptualised with the aim to match the aforementioned mechanical properties of native trachea. The use of 3DP technology has become more widespread within the field of bioengineering for customised tissue replacement or regeneration strategies for an array of organ systems. PCL was selected as the printing material due to its excellent printing characteristics. The thermoplastic nature of PCL and its low melting point temperature (60°C–70°C), makes it available for use for a wide variety of 3D printing methods. Furthermore, the mechanical properties of PCL has seen its widespread use in cartilage replacement in tissue engineering, and investigative studies in tracheal tissue engineering (Pfeiffer et al., 2008; Kundu et al., 2013; Hollister et al., 2016; Morrison et al., 2017; Les et al., 2019). Forgoing the solid polymer tubular design commonly used in recent tracheal scaffold fabrication attempts (Pfeiffer et al., 2008; Zych et al., 2012; Gao et al., 2017; O’Sullivan et al., 2019), two rings of PCL were fabricated into either complete 360° tubular ring or a 288° partial ring. The use of struts was hypothesised to not only generate pores for the collagen matrix to infiltrate within the 3DP framework, but also to provide a polymeric backbone with a degree of flexibility by generating space between the two rings. Also, layer-by-layer stacking of struts and rings were hypothesised to generate mechanical integrity and robustness—thus, producing framework designs that could potentially generate the necessary mechanical strength but also be flexible (Kaye et al., 2019).

To assess whether the 3D-printed tracheal framework designs could match the trachea’s anisotropic mechanical properties, all designs firstly underwent radial compressive testing (Figure 4A). Radially compressing samples is often performed on pipes for assessment of resistance to external compressive forces in cases where classical dog bone shaped samples are not feasible, as with curved tubular structures. Unconfined, uniaxial compression testing was utilised to assess the substrate stiffness of the 3D-printed frameworks radially between the different design features and the molecular weights of PCL. The resistance to compressive forces within the framework designs were greater than reported figures of human trachea which ranged from 1.06 to 1.08 N A-P and 0.55–2.32 N L (Huang et al., 2021), however, a study investigating the difference in mechanical properties within different large animal models and the influence on specimen length on mechanical properties reported figures within range of calculated values of our scaffolds, ranging from 2 to 5 N (Gao et al., 2017; Huang et al., 2021) in complete segments of tracheas tested at 4–8 rings in length at 50% deformation. For tracheal specimens radially compressed in both anterior-posterior and lateral testing positions, values of 2.38 N in canine and 2 N (A-P) and 2 N (L) both at 50% radial compression and 5 N at 40% in a goat trachea have been reported (Gao et al., 2017; Morrison et al., 2017). As already highlighted, the importance of matching the mechanical properties of the native tissue and implanted scaffold is vital, therefore while the mechanical strength of scaffold designs may be too high for human trachea, these designs could still be utilised for animal models. This also further supports the use of 3DP as it would allow for the adjustment of the designs to match mechanical properties of animal model with relative ease. Additionally, the resistance of mechanical forces of our scaffolds is much lower than other tissue-engineering attempts. 3DP PCL scaffolds fabricated for tracheal replacement reported values of approximately 23 N (She et al., 2021) and 30 N (Xia et al., 2019) of full length scaffolds, whereas electrospun tracheal scaffolds have generated values of 32.9 N (L) and 104 N (A-P) (Best et al., 2018) when displaced to 75% of luminal diameter, which are too mechanically stiff compared to native tissue. The 3DP frameworks generated in this study could therefore be beneficial for tissue integration as a function of their reduced mechanical resistance.

Although the trachea has excellent stability and radial strength in order to prevent collapse, it also needs to deform and quickly recover during breathing and coughing (Zhao et al., 2019). In an attempt to investigate similar durability of the PCL frameworks and also the influence of strut numbers on durability, cyclical loading and unloading was carried out for 250 cycles. Testing the designs in this manner would in theory increase the possibility of delamination occurring between the 3DP layers, leading to a loss of structural stiffness and ultimately failure of the scaffold design (Baumann and Hausmann, 2021). Positively, we found that all framework designs survived 250 cycles at 15% applied strain (Figures 4B, C). Cyclical testing of tracheal scaffolds has not been widely investigated, with researchers usually conducting simple lateral compressive or tensile tests only, however an ovine model tested under cyclical loading over 250 cycles under 15% strain reported a peak load of 0.06 N at cycle 1 and 0.04 N at cycle 250 (Mansfield et al., 2016). Through testing the different designs of our scaffolds, we were able to confirm that the 3DP PCL could withstand radial deformation over a large number of cycles without failure; furthermore, no visible failure sites or delamination were observed. A notable difference was observed between the two main design features, with the tubular design resistant to much higher radial compressive forces than the partial ring design. From the same testing regime of cyclical testing, the cyclical strain recovery was calculated to assess the percentage recovery of all the designs over 250 cycles of compressive loading. Notably, although a large difference in peak load was observed between tubular and partial ring designs, there was no significant difference in percentage recovery between the two main design features. This may be due to the high elastic modulus of PCL itself, which has been reported to correlate to the higher degree of crystallinity of 3DP PCL filaments and resultant higher elastic modulus and percentage recovery (Soufivand et al., 2020).

The final mechanical assessment assessed the flexibility of the 3DP designs by a three-point bending test. To reiterate, the trachea not only needs to radially deform to allow for narrowing and widening for passing of food through the oesophagus and coughing, but also needs to have a high degree of flexibility to allow for movement of the neck with ease (Rains et al., 1992; She et al., 2021). Furthermore, a highly flexible scaffold is less likely to dislodge when a patient suffers from a coughing fit, which has been an outcome *in vivo* and clinical trials of previous tracheal replacement efforts and a huge cause for failure (Grimmer et al., 2004; Azorin et al., 2006; Luo et al., 2013; Wood et al., 2014; Elliott et al., 2017). Designs T and PR generated flexural moduli of 0.13–0.26 MPa (Figure 4D), an order of magnitude lower than other tissue-engineering tracheal scaffold studies using 3DP PCL with a flexural modulus of 1.088 MPa (Park et al., 2018), thus making this study’s scaffold design much more flexible and compliant. However, the flexural moduli of rabbit trachea has been reported to range between 0.214 and 0.25 MPa (Pfeiffer et al., 2008; Best et al., 2018). Although no reported literature exists that has analysed the flexural moduli of human trachea, which is likely to be greater than smaller rabbit trachea, a study into the changes in mechanical properties of cell-seeded implanted tracheal scaffolds did find a 60% increase in flexural moduli 8 weeks post implantation from 1.71 to 2.84 MPa (Lin et al., 2008). Compressive and cyclical testing confirmed both second generation framework designs are still able to withstand radial compressive forces and are durable, therefore, higher flexural moduli could potentially be achieved in either design through matrix remodelling of cell-seeded scaffolds. Next steps would seek further surgeon evaluation of our designs, especially blinded evaluation, which would provide vital feedback on the scaffolds handling for future reiterations of the scaffold.

After establishing final 3DP parameters, designs, and mechanical properties for the tracheal scaffolds, we sought to investigate the biological properties of the composite scaffolds. The 3DP tubular PCL framework was successfully incorporated with CHyA films and slurry to fabricate composite reinforced CHyA-B scaffolds with ease and reproducibility, generating scaffolds with uniform matrix incorporation and tubular form factor. The mean pore size of the porous collagen sub-layer was analysed to investigate whether the incorporation of 3DP PCL within CHyA-B matrix had an effect, if any, on the pore size. The results indicated that the change in form factor of tubular CHyA-B scaffolds from flat 2 mm CHyA-B matrix used as *in vitro* respiratory models increased from 80 µm (O’Leary et al., 2016) to 180 µm. This increase in pore size may have been a result of the increase in mass of metal and overall different mould form factor of the tubular moulds in comparison to the smaller flat square moulds. This in turn influenced the freezing temperature within the mould, altering the freeze-drying profile of the −20 anneal cycle of the tubular scaffolds which impacted the pore size (Figure 6B). Moreover, the incorporation of the 3DP PCL framework into the CHyA-B matrix significantly increased the mean pore size from 180 μm to 290 µm (Figure 6A). Literature shows the ideal pore size for vascularisation is around 300–400 µm (Bai et al., 2010), therefore, the incorporation of 3DP PCL within the collagen matrix produced a composite scaffold with a sub-layer of collagen with a pore size ideal for cellular infiltration (Murphy et al., 2010) and vessel formation.

Having confirmed the feasibility of incorporating the 3DP PCL framework within the CHyA-B matrix and mechanically evaluated different designs for a tracheal replacement scaffold, we now sought to investigate the influence of incorporating the 3DP PCL framework on effective vascularisation *in vitro* and in the CAM model. Although collagen-glycosaminoglycan scaffolds are validated for *in vitro* and *in vivo* pre-vascularisation (McFadden et al., 2013), we wanted to ensure that the inclusion of the PCL reinforcement allowed for sustained vascularisation within the collagen matrix. The introduction of 3DP polymer fibres could impede cell-cell interactions, thereby decreasing vessel formation, and was a necessary risk to investigate. As such, the key objective of this study was to confirm the scaffolds’ ability to support cell growth of the *in vitro* co-culture system of HUVECs and hMSCs.

As with most 3D cultures, it was important to initially confirm cell viability in the PCL-reinforced scaffolds prior to any extensive analysis of angiogenesis or vascularisation. The composite scaffold exhibited comparable levels of cellular metabolic activity over 14 days of culture with no significant difference observed (Figure 7A), whereas levels of dsDNA present within the reinforced composite scaffolds were observed to be 2-folds greater than their non-reinforced counterpart (Figure 7B). The incorporation of the 3DP framework to CHyA-B scaffolds increased the mean pore size of the collagen sub-layer from 180 μm to 290 µm (Figure 6), and plausibly facilitated more cellular growth through augmented cell infiltration, nutrient inflow, and waste outflow (Murphy et al., 2010). Differences between the two viability assays may have been due to the differences in application of each assay. With the measurement of dsDNA content, the scaffold is broken down and cells are lysed whereas with measuring the cellular metabolic activity there could be limitations in how far the solution penetrates the scaffolds and thus provides limited measurements of the cells on the periphery of the scaffold. Finally, the presence of a high coverage of green viable cells on the surface of reinforced composite CHyA-B scaffolds was observed when stained with LIVE/DEAD^®^. Taken together, this further confirmed the biocompatibility of the scaffolds, with sustained cell growth and proliferation maintained with the co-culture model on reinforced composite CHyA-B scaffolds.

It is important to note that the composition of the polymer framework can also have a significant impact on viability, in addition to its effects on composite scaffold porosity. As previously discussed, PCL does not release cytotoxic degradation products into the cell culture environment, and therefore its biodegradation process does not impact cell growth (Sun et al., 2006; Brittberg, 2008) In contrast, other 3DP materials investigated for tracheal tissue replacement such as PLA (polylactic acid) and PGLA (poly(lactic-co-glycolic acid)) have biodegradation products that have been found to trigger an inflammatory response leading to a low pH-environment (Liu and Cao, 2007), and therefore causing an accelerated loss of structural integrity. For example, this was reported in a 3DP PLA tracheal scaffold which when implanted into the fascia of rabbits for pre-vascularisation, shrunk and deformed due to its instability whereas its PCL counterpart maintained its shape (Tsao et al., 2014). Thus, the addition of the 3DP PCL framework has shown to maintain cell viability while generating mechanical integrity.

The formation of vessel-like tubules was confirmed within reinforced composite CHyA-B scaffolds by several methods. The inclusion of the PCL framework to CHyA-B matrix has been shown to increase the pore size of the collagen matrix (Figure 6), thus it was imperative to assess if the change in pore size could impact vessel formation within the scaffolds. Firstly, confocal microscopy of fluorescently labelled cells within the scaffolds demonstrated the presence of vessel-like tubules for up to 14 days of culture (Figure 8). Notably, we also confirmed that the tubule-like structures were characterised by the lining of HUVECs (CD31) with hMSCs (α-SMA) in direct contact by staining with cell specific markers (Figure 9). As with the previously established model (McFadden et al., 2013), a similar trend was observed, with the strongest vessel formation occurring on day 10 of culture followed by noticeable vessel regression at day 14. As is the issue often associated with long term static *in vitro* culture of microvascular networks, disengagement of cells can take place, resulting in structural regression of the networks, thus limiting the extent to which vessel development within a scaffold can take place *in vitro*. However, once implanted in a dynamic environment *in vivo*, recruitment of host perivascular cells can promote further stabilisation of blood vessels (Montaño et al., 2010).

This possibility was bolstered by the analysis of angiogenic marker expression in cell-seeded scaffolds. No statistical difference seen between the CHyA-B matrix and the reinforced composite CHyA-B scaffolds with regards to expression of angiogenic genes (Figure 11) and proteins (Figure 10), indicating parity in vascularisation potential. The protein bFGF (also known as FGF-2) and its counterpart FGF-1, are potent pro-angiogenic markers which promote the proliferation and differentiation of endothelial cells (Lee et al., 2000; Oladipupo et al., 2014). The expression of both VEGF and bFGF is dynamic during vascularisation (Bautch, 2012; Brundo et al., 2013). The stabilising of KDR (also known as VEGF receptor 2) and FLT 1 (also known as VEGF receptor 1) expression at day 14 but the increase in expression of ANG 1 and its receptor TEK 2 could suggest the maturation of vessel-like structures occurring within the scaffolds from early vessel sprouts to more mature vessel tubules. KDR and FLT-1 are major signal transducer for angiogenesis, promoting vessel formation (Helfrich and Schadendorf, 2011) and when the pro-angiogenic VEGF binds and activates signalling via its receptor KDR on endothelial cells, extracellular matrix degradation, tip cell migration and endothelial proliferation are promoted to form new immature vessel sprouts. Whereas ANG 1 is a stimulator of vessel growth and along with its receptor TEK 2 promotes vessel stabilisation (Lambert et al., 1991; Roberts et al., 1997). However, the upregulation of ANG 2, a marker that causes disruption of vascularisation and destabilisation (Helfrich and Schadendorf, 2011) would suggest the co-culture model is not able to hold up angiogenesis at day 14 as confirmed previously (McFadden et al., 2013) and as shown in fluorescently labelled cultured scaffolds on day 14 imaged samples (Figure 8).

In the first step of *in vivo* validation of the reinforced scaffolds, their vascularisation potential was further validated using a CAM model, with an increased area of vascularisation measured within the reinforced composite CHyA-B scaffolds (Figure 12; *p* = 0.1929). As with the cellular proliferation, the increased pore size of the reinforced composite CHyA-B scaffolds may have allowed for greater blood vessel infiltration, as a mean pore diameter of 300–400 µm has been found to be ideal for vascularisation (Bai et al., 2010). Taken together, not only did the presence of 3DP PCL framework not have any detrimental impact on vessel formation, but it possibly may even enhance vascularisation within collagen-based scaffolds.

Indeed, several approaches to vascularise tracheal scaffolds have been investigated elsewhere in an attempt to accelerate neovascularisation of tracheal grafts, such as the application of angiogenic factors such as VEGF and human recombinant erythropoietin (hrEPO; 294,295). Although preliminary results showed promise in vascularising grafts, the maintenance of working concentrations of growth factors post-implantation has not yet been investigated (Tan et al., 2006). Furthermore, this approach relies of efficient host response and vascularisation may not occur in time to allow for graft survival. Another more widely-investigated approach has been pre-vascularising tracheal grafts through the means of implanting the graft into the patients forearm or omentum prior to implantation into the defect site (Lendlein et al., 2001; Delaere et al., 2019). The use of an initial heterotopic vascularisation period elsewhere in the body, prior to tracheal transfer, has been shown to generate a clinically significant blood supply (Kalathur et al., 2010; Kim et al., 2011; Udelsman et al., 2018; Li et al., 2019). However, such a strategy can take several weeks––or even months––to fully establish a vascular network and is therefore not adequate in emergency situations (Maughan et al., 2017). On the other hand, the means of pre-vascularising the reinforced composite CHyA-B scaffolds via the co-culture system, which we have shown can successfully vascularise *in vitro*, can establish a network of vasculature within the scaffolds to ensure success of graft acceptance into defect site within shorter time frames, as shown by previous success within our lab (McFadden et al., 2013). These mechanically reinforced pre-vascularised scaffolds can now be evaluated for their impact on enhancing epithelisation.

While this study has successfully achieved its objectives and developed a novel tubular scaffold for tracheal replacement, the lack of degradation data of our 3DP framework design, is a principal limitation. Although there are *in vivo* degradations studies on PCL that can provide an estimate 2–3 years degradation rate (Sun et al., 2006); the speed at which PCL breaks down is largely dependent on its surface area, geometry and molecular weight, all of which differ in studies. Completing a comprehensive degradation study would require a long-term animal study necessitating a large quantity of animals, which is unfortunately out the scope for the length of this research project. Reducing the number of animals required could be achieved by developing a non-invasive procedure to measure degradation within animals without requiring euthanasia, such as using micro-CT. Moreover, 3D-printed framework designs were mechanically assessed in a dry state due to the limitations of the testing machine, however, more appropriate assessment would be done under a hydrated state to mimic physiological conditions. Under hydration, PCL rate degradation would differ as the solution would enhance the hydrolytic breakdown of its ester bonds and thus allow for a more accurate measurement of each design’s response to mechanical load. In particular, fatigue testing performed in a hydrated state over a longer rate of cycles such as for spinal implants (ISO 12189:2008) would provide vital information of the scaffolds durability. Moreover, tracheal tissue is viscoelastic, as this study has successfully established the foundation for a mechanically compliant and flexible scaffold, future refinement of the scaffolds can look to characterise its viscoelastic features.

## 5 Conclusion

Overall, we were successfully able to fabricate a composite tissue engineered tracheal substitute that mimics the reported mechanical properties of native tracheal tissue and confirmed the incorporation of 3DP PCL framework within the CHyA-B matrix did not impede on vessel formation using an *in vitro* culture model. A battery of tests was developed to characterise the 3DP designs by measuring mechanical strength, robustness, and flexibility, all of which are integral tracheal properties. This has enabled us to establish two main designs which provide a range of mechanical properties close to animal models, allowing us to streamline a design in the future when more data becomes available on the exact mechanical properties of the trachea. The use of PCL in tissue engineering, in particular tracheal reconstruction, is becoming more widespread. Although PCL exhibits biocompatibility it is less biocompatible than natural polymers, and as previously discussed, tracheal scaffold attempts with PCL alone have induced foreign body and inflammatory reactions. However, the ease of printability and excellent mechanical properties of PCL are substantial advantages, therefore we combined the 3DP PCL with freeze-dried CHyA-B matrices to create a composite reinforced scaffold, which was confirmed to support the growth of cells *in vitro*. The successful pre-vascularisation of our reinforced composite CHyA-B scaffold lends itself resolving a major hurdle in the advancement of tracheal scaffolds. By addressing both the mechanical and physiological requirements of a tracheal scaffold, this work has begun to pave the way for a new therapeutic option to resolve the shortcomings of current treatment options, potentially improving patient outcomes.

## Data Availability

The raw data supporting the conclusion of this article will be made available by the authors, without undue reservation.
